# Using qualitative data to build causal pathways on perceived associations between poor menstrual health and hygiene and harms among schoolgirls in western Kenya

**DOI:** 10.3389/frph.2026.1931799

**Published:** 2026-10-07

**Authors:** Ellie Ansell, Israa Saleh, Garazi Zulaika, David Obor, Anna Maria van Eijk, Kelvin Oruko, Sandra Oketch, Penelope A. Phillips-Howard, Linda Mason

**Affiliations:** 1 Department of Clinical Sciences, Liverpool School of Tropical Medicine, Liverpool, United Kingdom; 2 Department Applied Health Sciences, School of Health Sciences, College of Medicine and Health, University of Birmingham, Birmingham, United Kingdom; 3 Safe Water and AIDS Project, Kisumu, Kenya; 4 Kenya Medical Training College, Nairobi, Kenya; 5 Centre for Health Services Studies, University of Kent, Canterburty, United Kingdom; 6 Centre for Global Health Research, Kenya Medical Research Institute, Kisumu, Kenya

**Keywords:** causal pathway, infections, Kenya, menstrual health and hygiene, pregnancy, qualitative, school absence, schoolgirls

## Abstract

**Background:**

Poor menstrual health and hygiene (MHH) is a global public health concern, particularly in low- and middle-income countries. It can result in adverse health and educational impacts. Narratives from baseline focus group discussions (FGDs) conducted during a pilot intervention study in rural western Kenya were explored to reveal perceived associations.

**Methods:**

Transcripts of 19 FGDs involving 87 post-menarche schoolgirls, 47 parents, and 46 teachers were analysed thematically to identify perceived causal links between menstrual issues and health and educational outcomes. Quotes illustrating these perceived cause-and-effect relationships were logged and synthesised into a series of pathways, from which an overarching causal pathway model was produced.

**Results:**

Poverty underpinned poor MHH, characterised by the inability to afford products, the use of inadequate products, and the prolonged use of products. Key outcomes included menstrual issues, menstrual accidents (e.g., leaking), psychosocial distress (e.g., humiliation), poor educational outcomes (e.g., school absence), transactional sex, adolescent pregnancy, and infections (e.g., localised or reproductive tract infections). Poor MHH outcomes were often perceived as “nodes” or drivers that led to further distal outcomes. Four pathways emerged, all stemming from poverty. In pathway 1, poverty led to poor educational outcomes via inadequate menstrual materials, menstrual accidents, and psychosocial stress. In pathway 2, poverty led to poor educational outcomes via inadequate menstrual materials and physical discomfort. In pathway 3, poverty caused infections through the use of inadequate materials. In Pathway 4, inadequate materials resulted in transactional sex.

**Conclusion:**

Four perceived pathways linking poverty and poor MHH to an endpoint were used to develop a single overall causal pathway. The most frequently perceived outcome was school absence, with pregnancy and school dropout noted much less frequently. As poverty, the perceived underlying cause in this pathway, is a complex target, focusing interventions on proximal perceived key nodes in the pathway, such as poor MHH, would be of value.

## Introduction

Each day, some 300 million females manage their menstruation worldwide, yet many are unable to do so in a healthy and dignified manner due to inadequate access to appropriate menstrual products and facilities (1, 2). Menstrual health and hygiene (MHH) encompasses the essential resources, services, and support systems required for effective menstrual management. This includes access to accurate menstrual education, social support, affordable and safe menstrual materials, and effective water, sanitation, and hygiene (WASH) infrastructure (3, 4). Beyond hygiene, the concept of MHH is associated with broader systemic factors, intersecting menstruation with education, equity, empowerment, and overall physical and mental health and well-being (3). Socially, women and girls require safe and stigma-free supportive environments and systems to manage menstruation without fear or anxiety (4). However, several limitations and significant barriers persist that prevent the safe and effective management of MHH for girls and women in low-income settings (1, 3, 5), which can lead to adverse adverse health outcomes such as reproductive tract infections (RTIs) and psychosocial distress (6, 7).

Achieving good MHH is fundamental to promoting equality, rights, and human dignity for those who menstruate (8, 9). The Sustainable Development Goals (SDGs) do not explicitly target MHH, which may contribute to limited prioritisation and investment (3). Although SDG 6.2 (WASH) has potential relevance to MHH, its indicators are gender-blind and fail to capture the specific needs of those who menstruate (3). To address this gap, experts have developed global monitoring frameworks to measure MHH progress (10).

Qualitative and quantitative studies have explored the impacts of ineffective and inappropriate MHH (1, 11, 12). However, a broader perspective on understanding the perceived relationship between drivers of poor MHH and consequent harms can provide valuable insights. For example, what do girls, parents, and teachers experience and understand about the causes of poor MHH and their effects on health, well-being, and schooling for girls?

Mapping a causal pathway for MHM in sub-Saharan Africa (SSA) is important to move current research beyond simply documenting associations to explaining how and why different social, economic, environmental, and health factors influence MHM (1), its antecedents, and downstream consequences. Consequently, there is growing recognition that effective menstrual health programmes require integrated, multi-sectoral approaches that combine menstrual products, WASH services, education, social norm change, and supportive policies (13–15). A causal pathway can contribute by providing a holistic picture that informs the causes, needs, and appropriate timing of such interventions.

This study utilises qualitative data to (1) examine the perceived factors associated with poor menstrual health and associated health and schooling problems and (2) map the perceived causal pathways between perceived poor MHH drivers and harmful outcomes, utilising data gathered from schoolgirls, parents, and teachers in western Kenya.

## Materials and methods

This secondary study aims to utilise qualitative data to examine the perceived effects of poor menstrual hygiene on the health and schooling of adolescent schoolgirls in western Kenya and to map causal pathways. The objectives were to:

1. Conduct thematic analysis on the qualitative data to identify perceived factors associated with poor menstrual health and associated health and schooling problems; and
2. Utilise narratives on perceived associations to map, chronologically, where these factors are perceived to lie within emerging causal pathways.

### Study design

This study is a qualitative analysis of secondary data collected as part of a wider study, The “Menstrual Solutions” Study (MSS) (described in full elsewhere, Phillips-Howard et al., 2016), using transcripts from focus group discussions (FGDs) involving primary schoolgirls, parents, and teachers.

### The “Menstrual Solutions” study

This was a three-arm, single-site, cluster-randomised controlled pilot study conducted among primary schoolgirls aged 14–16 years in Siaya County, western Kenya, between 2012 and 2014. Thirty primary schools were randomised into three arms: menstrual cups, sanitary pads, or usual practice. The study examined the cultural acceptability, use, and safety of menstrual cups and menstrual pads compared with usual menstrual practices (6, 16). In addition to quantitative data, qualitative data were collected at three different time points to provide contextual information that helps explain the study outcomes. For the present study, we used data collected at baseline, prior to any intervention. This dataset from three participant groups has not been analysed together previously, nor developed into causal pathways. Although the data were collected some time ago (2012), we noted a lack of studies developing menstrual causal pathways. Furthermore, the issues emerged remain highly relevant to the current MHH context, suggesting a lack of progress.

### Study setting

The study was conducted in Siaya County, a rural area in western Kenya near Lake Victoria and 40 km from Kisumu, the third-largest city in Kenya. The study site was part of a health and demographic surveillance system, which enhanced participant follow-up. The study area consists of predominately Luo-ethnic communities whose primary income comes from subsistence farming and fishing (17). The county is demographically young, with 41.3% of the population under the age of 15 years (18). The site was selected for the pilot study primarily due to the presence of a Health and Demographic Surveillance Survey site and is an impoverished area, with emerging evidence—since corroborated (11, 19)—indicating that young adolescent girls may resort to transactional sex to obtain sanitary pads. Correspondingly, HIV incidence rises sharply among adolescent girls (6).

### Participant recruitment

We used random sampling to select six of the 30 schools for FGDs. We anticipated that six FGDs per participant group would provide sufficient data to meet our study aim without being overly burdensome for the schools and individuals already participating in the trial. We intended to recruit additional schools as necessary if we did not reach data saturation for any participant group (i.e., schoolgirls, parents, and teachers). For each participant group, we used convenience sampling, assuming that contribution and views of all participants would be relevant to our study needs. Members of the qualitative research team visited each school to obtain permissions from headteachers and assistance with the logistical aspects of recruitment. Information about the study and its involvement was provided to girls who consented to the main study, their parents, and all teachers from these selected schools. When more individuals wished to participate than were needed, we selected on a first-come, first serve basis. At the time of FGDs, all participants were taken through the informed consent process before signing the consent and for the girls, assent forms.

### Focus group approach

FGDs were chosen as the data collection method to incorporate a wide range of viewpoints and to stimulate discussion with minimal input from the moderator (20). A discussion guide was developed by the research team around key topic areas of interest, informed by the study's key objectives and current literature. The guide was individualised for the three participant groups while comprising considerable topic overlap to facilitate comparisons. It covered experiences and understandings of menstruation and menarche, probing into issues such as hygiene, menstrual practices, risks, and the effects of menstruation on eduction, as well as barriers and facilitators to menstrual health and hygiene practices. Three young local females trained in qualitative data collection and ethical practices served as moderators and note-takers, working in alternating pairs per FGD. Each FGD was conducted using a mixture of Dholuo and English, as participants preferred. The moderator led the FGDs, while the note-taker captured key points of the discussion, verbal gestures, and group dynamics. All FGDs were conducted on school premises in a quiet, private room. Each discussion began with introductions, a project description, and a discussion of ground rules and informed consent procedures. FGD sessions lasted 1–2 h and were audio-recorded with consent. Participants were assigned identification numbers, e.g., Participant 1 (P1), to prevent disclosure of names. The note-taker recorded, where possible, the identification numbers of the participants who spoke in the FGDs. The audio recordings were translated (as needed) and transcribed verbatim into English by a trained Kenya Medical Research Institute (KEMRI) research staff member and subsequently verified for accuracy by a second staff member. To ensure that data saturation had been reached, FGDs were continually reviewed during data collection, and we observed multiple similarities in the narratives expressed, with little new data emerging.

### Data analysis

#### Thematic analysis of FGD transcripts

The FGD transcripts were initially analysed using thematic analysis, following Braun and Clarke's thematic analysis framework, whereby FGDs were examined individually by participant type to gain maximum perspective and insight into similarities and differences in menstrual health experiences (21). This provides a clear framework to work upon and analysis beyond the point of description, providing a deeper understanding of the underlying assumptions and mechanisms within the transcripts than other thematic frameworks, which is key to understanding causal pathways (21). An inductive approach was used, whereby the themes and pathways emerged from the data.

The English transcripts of the FGDs were read several times by members of the research team (EA, PP-H, and LM) to ensure familiarisation with the data. Once familiarised, initial considerations for the coding framework were generated by EA, who highlighted and annotated the transcripts with code ideas around the perceived menstrual issues noted by participants. This was done manually in Word. Frequent discussions were held with PP-H to discuss, interpret, and reach consensus on data meanings. All assigned codes were reviewed and verified by PP-H to generate themes on the problems associated with poor menstrual management. Notes and discussions were recorded to ensure interpretative accuracy and to confirm that chosen illustrative quotes accurately representated the context. Themes were reviewed by team members to eliminate overlap, with emergent “causal” and “outcomes” identified as the two key themes, with relevant subthemes incorporated as identified. A coding framework was then built by EA, featuring illustrative quotes for the codes. Analysis incorporated comparison of views, contradictions and agreements, strength of feeling, and phrasing used. A narrative was then constructed using a selection of quotes to exemplify each analytical point.

#### Building causal pathways

Participants stated a combination of perceived causes and outcomes during discussions of MHH, supporting the exploration of these factors to inform our research on causal pathways. To develop the perceived causal pathway, we eliminated any quotes that did not include causal reasoning or linkage to a perceived associated outcome, as noted by the participant. Each perceived association between cause(s) and outcome(s) was logged into a Microsoft Excel spreadsheet, as illustrated in Figure 1 (see the Results section). The perceived “emergent cause” stated in the quote was noted, alongside other perceived “emergent” outcomes. The quotes were then arranged in a cascading sequence to demonstrate the hypothesised linkages between the cause(s), outcome(s), and reported menstrual issues, ensuring chronological coherence (see Supplementary File S1 for all relevant quotes).

**Figure 1:**
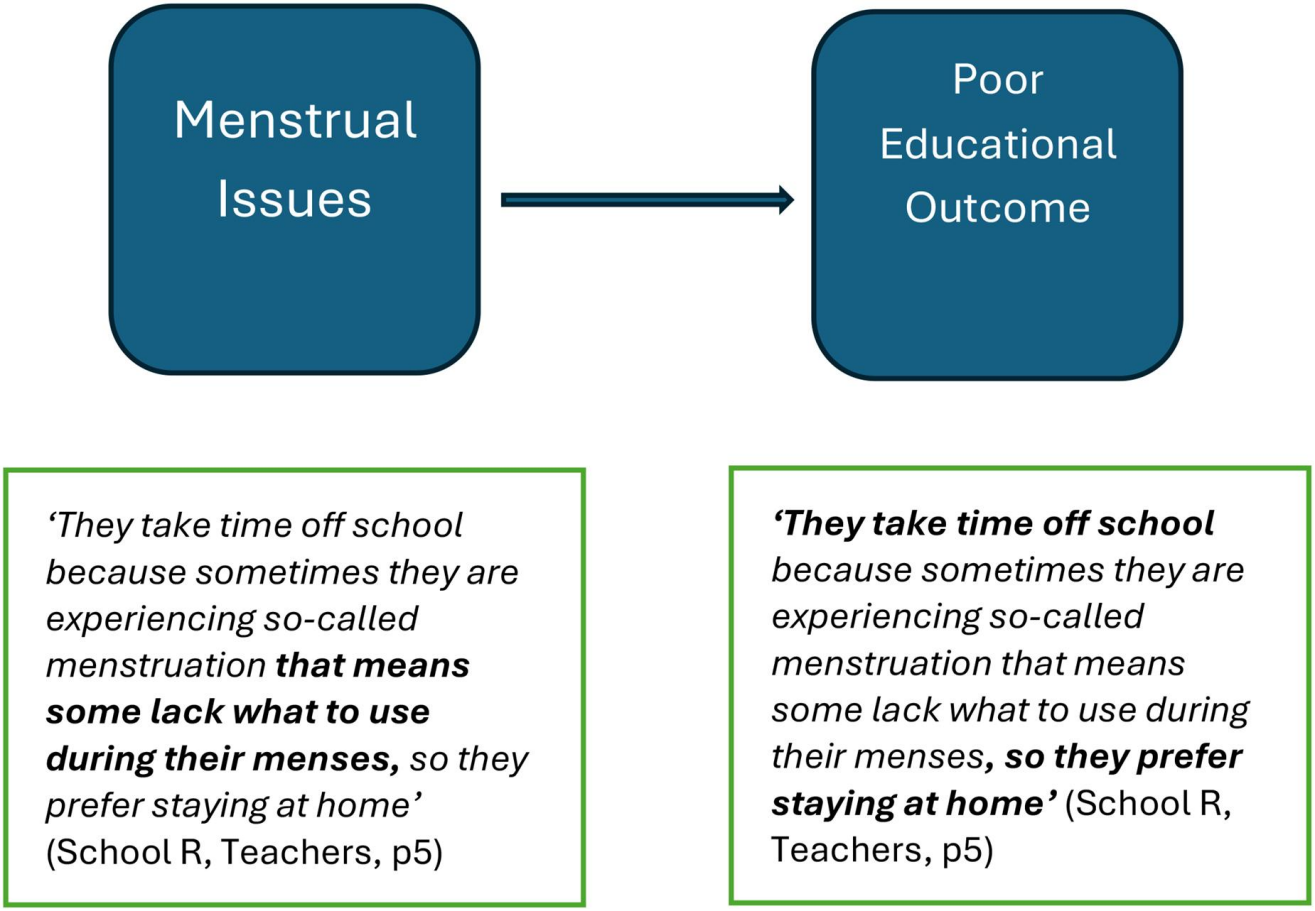
Part pathway 1.

A critical realist approach (22, 23) was then used to establish the resulting causal pathways. Quotes were examined for cause-and-effect associations expressed or noted by a participant to identify perceived causal factors associated with previously identified emergent outcomes of menstrual issues. In addition, a retroductive analysis was utilised to examine cause-and-effect configurations and to identify mechanisms capable of producing the narratives, for example, perceived menstrual issue-related outcomes, the causal mechanisms, and the reasoning behind the lack of menstrual products for girls and women (24). Quotes were mined for demi-regularities (i.e., common or frequently expressed causal factors or mechanisms that account for multiple observed effects) to establish the perceived causal pathways (25). Participants expressed varying demi-regularities across the causal pathways, contributing different elements to the pathways. As illustrated in Table 1, words were highlighted to demonstrate which components of the quote corresponded to specific major themes or subthemes.

**Table 1:** Example of assigning quotes.

School	Participant	Quote	Emergent outcome	Emergent causes	Other emergent outcomes	Notes
School J	Parent—P1	I think that if we can afford to buy them pads they will just go to school because most of them take time off going to school depending on the method they use. They just use anything they get at home which may leak even in class making them feel embarrassed but when they use pads they are very comfortable and go to school as usual	School absence	Inability to afford pads	Menstrual accidents and psychosocial stress	N/a

The thematically identified causes and outcomes were assigned as “nodes” within the pathways. Quotes were pasted along the various pathway nodes they represented. The specific part of the quote corresponding to that node was highlighted to represent how the quote builds into the pathway.

Based on the nodes identified by participants, the pathway components were delineated into perceived root causes, early drivers, intermediate drivers, and outcomes. The reasoning for these delineations was as follows. For the root causes (i.e., poverty), no participant identified any issue that preceded this. The early drivers were identified as directly stemming from the root cause but often leading to further consequences or outcomes. The intermediate drivers could lead to other intermediate drivers (e.g., menstrual leakage leading to psychosocial stress) before ultimately culminating into a subsequent outcome. The outcome was the terminal point at which no participant identified any additional consequences.

Where issues were identified without an associated cause or did not contribute to an additional driver or outcome, as indicated by participant quotes, they were not included in the pathway (e.g., odour, menstrual pain).

The study's trustworthiness was established using systematic validation checks. EA maintained an audit trail of the analytical process to ensure dependability and confirmability, with oversight and regular debriefing by PP-H. To ensure credibility, we employed investigator and participant triangulation; thematic consistency was observed across all FGDs, and all investigators agreed on the findings. Finally, the potential transferability of both our initial analysis and causal pathways to similar settings in SSA is supported by the broader literature, including a primary analysis of data on girls and parents (16).

## Results

### Participant information

Nineteen FGDs were conducted across the three different participant groups (detailed in Table 2). A total of 180 informants were included, comprising 87 girls, 47 parents, and 46 teachers.

**Table 2:** FGD composition.

Participant group	Number of FGDs	Total participants
Girls (aged 14–16 years)	8	87
Teachers	5	46
Parents	6	47

The number of participants in each FGD ranged from 5 to 12. Teacher FGDs included both male and female teachers, of whom 60% were male. Demographic data for parents were not recorded. The girl were aged 14–16 years. In one school, high student interest resulted in more volunteers than the maximum threshold of 12 participants, resulting in the formation of two separate FGDs.

The themes were categorised into perceived “Causes” and “Outcomes.” Girls spoke about their personal experiences, whereas parents and teachers focused on the experiences of their daughters and students, respectively.

#### Causes

##### Poverty

Perceived causes were underpinned by a single major theme: poverty. Three subthemes emerged describing how poverty worked as a causal factor in menstrual health and hygiene issues (see Table 3**)**.

**Table 3:** Emergent causes.

Causes—major themes	Causes—subthemes
Poverty	Inability to afford menstrual products/materials
	Lack of sufficient or appropriate menstrual products
	Prolonged use of menstrual products

##### Inability to afford menstrual products

Parents and teachers frequently noted that the prevailing poverty affected the ability to afford any or enough menstrual products to meet the needs of their daughters and students. They noted that sanitary pads were too expensive and therefore unaffordable for most families.

Even during menstruation when they cannot afford the attires… (School O, Teachers, P2)

 …back at home we cannot afford sanitary towels… (School Q, Teachers, P1)

…you cannot buy her pads since they are expensive and most parents cannot afford. (School M, Parents, P2)

Girls also echoed the narrative of unaffordable pads, linking their inability to buy menstrual materials to the financial struggles of their parents.

Some can tear mattresses to use if their parents cannot afford to buy them things to use to manage their menses. (School H, Girls, P6)

My mum cannot afford to buy me what to use during menstruation… (P9, School H, Girls)

##### Lack of adequate menstrual products

Participants across all groups reported that girls used inadequate materials to manage menstruation. A wide range of inadequate and inappropriate menstrual materials were described, including fragments of mattress or blankets, scraps of cloth, rags, and grass.

Some use sacks. (School E, Girls, P9)

…they will just use their rags. (School P, Teachers, P5)

They use blankets… (School J, Parents, P6)

##### Prolonged use of menstrual products

Not having enough menstrual products led girls to state that they used their menstrual products for longer than they ought or wanted to, a situation reported by all groups of participants. Another reason they reported for prolonged use was that girls did not have the opportunity or wherewithal to change their material and therefore kept the same pad, or other material, in place throughout the day. This included while at school.

When you use old rags …. some use them from morning to evening… (School P, Teachers, P1)

… they put them on from morning till evening … (School L, Parents, P1)

#### Outcomes

The following major themes and subthemes emerged as outcomes from the described causes above (see Table 4).

**Table 4:** Emergent outcomes.

Outcomes—major themes	Outcomes—subthemes
1. Menstrual issues	• Discomfort/pain/bruising • Odour
2. Menstrual accidents	• Leakage • Dropping materials
3. Psychosocial distress/mental health	• Shame, embarrassment, fear, and general anxiety
4. Poor educational outcome	• Reduced school engagement/concentration • Self-absenteeism from school • School dropout
5. Transactional sex	
6. Adolescent pregnancy	
7. Infections	• Reproductive tract infections • Sexually transmitted infections

##### Menstrual issues

###### Discomfort

Girls across the different schools noted feelings of discomfort from using inappropriate items, noting pain, chafing, or burning sensations from bruises, cuts, rashes, and wounds,

The more you continue using grass, the more it severs the wound in the vagina… (School A, Girls, P1)

…thighs get irritated, you can't even walk comfortably. (School G, Girls, P4)

…if you use damp cloths you might start itching then it turns to a wound. (School H, Girls, P1)

It was often noted that bruises developed first, from friction with unsuitable items, which then developed into wounds over time. Girls and their parents reported that friction and irritation occurred because girls did not change during school hours,

When they wear clothes … put them on from morning till evening, so they get bruises on their thighs due to friction and the cuts are painful! (School L, Parents, P1)

###### Menstrual pain

Similarly, menstrual pain or feeling unwell was also occasionally noted by parents as a cause of girls' absenteeism,

…take time off school that she feels lower abdominal pain for even three days. (School J, Parents, P1)

###### Odour

Odour due to inadequate materials was noted by teachers and parents but not by girls,

…some of them were using cloths…the smell is too much… (School Q, Teachers, P8)

…you are still fresh unlike blanket or cloth that will make you have a foul smell. (School M, Parents, P7)

##### Menstrual accidents

###### Leakage

Girls frequently reported soiling their clothes due to a lack of menstrual materials, the use of inappropriate materials, or the prolonged use of their absorbent materials.

…I've only worn my underwear, so when I get up, my dress is already soiled. (School D, Girls, P4)

Sometimes your cloth is soiled… (School C, Girls, P7)

Having stained garments, commonly school dresses, was another variation of menstrual accidents, reported by all groups,

…their clothes are stained with blood… (School, J, Parents, P5)

…their uniforms will be stained. (School S, Teachers, P12)

…your clothes will be blood stained… (School D, Girls, P3)

###### Loose materials

Menstrual materials dropping out while wearing them were also noted by participants across all groups. This was usually associated with the use of improvised materials and disposable sanitary pads.

…cloths might come out… (School L, Parents, P1)

…when she jumps up and down the rags might fall off. (School S, Teachers, P4) and

…sometimes they didn't place it very well, so maybe it can fall when she is running. (School G, Girls, P5)

##### Psychosocial distress/mental health

Girls reported experiencing emotions such as shame, embarrassment, fear, and general anxiety when during menstruation, a psychological impact of which parents and teachers were aware. Participants across all groups noted that these stressors were mainly caused by peers laughing or teasing following visible menstrual leaks, or by the fear that such an incident might happen.

I am embarrassed … I've only worn my underwear, so when I get up, my dress is already soiled. (School D, Girls, P4)

…when you stain your clothing …. people will start laughing at you and you be ashamed. (School H, Girls, P5)

…other pupils were laughing, and the dress was like wet… (School P, Teachers, P6)

…she is not happy because she feels it can leak in class…she will be embarrassed. (School M, Parents, P8)

##### Poor educational outcomes

###### Reduced school engagement/concentration

Reduced concentration and diminished classroom engagement were commonly highlighted as consequences of inadequate menstrual management. Girls admitted that they could not concentrate in class due to the fear of staining or soiling their clothes while in class,

… blood stained, and the teacher will see hence you don't concentrate. (School D, Girls, P3)

Teachers also noted a reduction in girls’ engagement in school activities.

Even during the games period, they can be excused not to jump because if you have worn a rag and it will fall down*.* (School O, Teachers, P1)

…when you use the old rags, they get infections…you cannot concentrate during class … learning will not take place. (School P, Teachers, P1)

###### School absenteeism

Teachers and parents noted that girls missed school during menstruation, primarily due to a lack of adequate menstrual products. Although girls themselves did not usually volunteer information about their absence, they acknowledged that others missed school for this reason. Only one participant admitted that she had missed school.

They just stay at home because they don't have proper materials to manage their menses… (School M, Parents, P7)

…they cannot afford the attires to use during this period, some of them will not come. (School O, Teachers, P2)

Teachers also noted that the onset of menstruation was a common cause of school absenteeism among girls.

…she is absent and maybe it is a girl who has never been absent… you realize that she is absent because of the menses… (School R, Teachers, P10)

Parents noted that menstrual pain or feeling unwell due to pain or discomfort from using traditional items contributed to girls' absenteeism.

Sometimes she doesn't go to school due to bruises she got from using blanket. (School I, Parents, P10)

###### School dropout

School dropout was rarely discussed and, aside from one instance, was not identified as a direct consequence of menstruation, but rather as an indirect outcome mediated by pregnancy. Two girls from different schools shared that girls could drop out of school due to pregnancy,

…if not careful they can become pregnant thus dropping out of school.” (School A, Girls, P3)

Two teachers from one school noted that girls dropped out due to pregnancy and marriage,

‘M—So what happens when they conceive?—“They will drop out of school” (School P, Teachers, P4)

“…parents do not allow them to come back to school… after a short period of time you hear they are married, even as young as possible …” (School P, Teachers, P2)

##### Transactional sex

Girls, parents, and teachers all mentioned that girls engaged in transactional sex for money to buy menstrual products such as pads. Occasionally, pads were bought by males and given as a prize in a transaction. While no girls admitted that they themselves had boyfriends or sexual partners, they reported knowing “other” girls within the school who maintained sexual partners and that these girls would engage in sexual activity in exchange for menstrual products, money, or other items.

He can buy for you sanitary towels. (School A, Girls, P1)

…the girls might start having sex with men who give them money… (School K, Parents, P5)

Some people exchange sex for money. The money given is used to buy pads… (School H, Girls, Px)

“M—Yes what do they get from their boyfriends?” … “The pads and cash” (School P, Teachers, P2)

##### Adolescent pregnancy

Participants noted that some girls experienced pregnancy, although discussions surrounding this topic were very limited. While some attributed pregnancy to relationships with boyfriends, they did not specify whether those relationships were transactional.

…disadvantage is that he can impregnate you if you indulge in sexual intercourse. (School B, Girls, P12)

She will go look for this money from then men, and that's how they can end up with the unwanted pregnancies. (School L, Parents, P2)

##### Infections

###### Reproductive tract infections

Girls and teachers noted infections arising from the use of poor-quality materials, although the type of infection or symptoms was not disclosed or discussed.

You will again use the same grass to manage your menses. It will make you sick. You can get a variety of infections (School A, Girls P3)

One girl referred to the infection as a “…*problem in the vagina*” (School G, Girls, P9) resulting from the use of mattress fragments to manage menstruation, but this was not discussed further. Others noted infections developed in association with specific types of menstrual materials used, with “*overstaying*” of materials similarly identified as a contributing factor.

Okay when you use old rags, they get infections… (School P, Teachers, P1)

I am not a doctor but I think they stay long with whatever they use so that can cause infections. (School K, Parents, P5)

###### Sexually transmitted infections

Unlike RTIs, girls and teachers discussed sexually transmitted infection (STIs) as an outcome related to engagement in sex but not directly from menstrual materials, nor directly resulting from transactional sex. Parents did not discuss STIs. Three girls from separate schools noted that an STI resulted after they had engaged in sexual activities.

Some people do say if you have started engaging yourself in sexual behaviour you might be infected with HIV or even Sexual Transmitted Diseases. (School H, Girls, P2)

He can sleep (having sex) with you then he infects you with disease. (School C, Girls, P1)

### Development of causal pathways

Four pathways emerged when linking participant perceptions of causes (key drivers) to outcomes. These are presented below with exemplar quotes.

#### Pathway 1: poverty leading to poor educational outcomes via inadequate menstrual materials, leakage, and psychosocial distress

Poverty was frequently identified as the key societal factor driving girls to use inadequate menstrual materials or engage in prolonged use. This “immediate” or proximal segment on the pathway was evident, strongly linking the inability to afford menstrual products with increased use of poor-quality materials, as noted by girls, parents, and teachers alike. All participant groups commonly reported that using inadequate materials or prolonged use could cause leakage and/or staining. Similarly, all participants reported that this leakage, or the fear of it, caused girls' psychosocial stress, illustrating that leakage functioned as both an outcome and as a driver for subsequent effects (such effects can be considered as “nodes” along the pathway). Girls reported feeling embarrassed, scared, ashamed, or fearful of others, specifically classmates and teachers, noticing and teasing them when leakage happened. Parents and teachers acknowledged that girls experienced these emotions. A commonly perceived distal outcome associated with leakage and the resulting psychosocial stress was poor educational outcomes. Girls, parents, and teachers observed that schoolgirls experienced absenteeism during menstruation, as depicted in Figure 1. Only one teacher highlighted that an incident of leakage and subsequent humiliation led a girl never to return to school (i.e., resulting in dropout). Other quotes lacked clarity as to whether the absence was permanent or temporary. No other teacher, parent, or girl overtly linked poor school attendance due to menstrual issues with school dropout. Repercussions from these poor educational outcomes were not discussed or explored further, thus concluding this pathway.

The following two examples illustrate common nodes related to menstrual issues that link cause and outcome on this pathway. Figure 1 illustrates how some nodes were missed out i.e., “leakage*”* and “psychosocial stress” but followed the direction and causal links, illustrative of the frequent observations made along this pathway. Figure 2 shows the full pathway with a quote illustrating all nodes.

**Figure 2:**
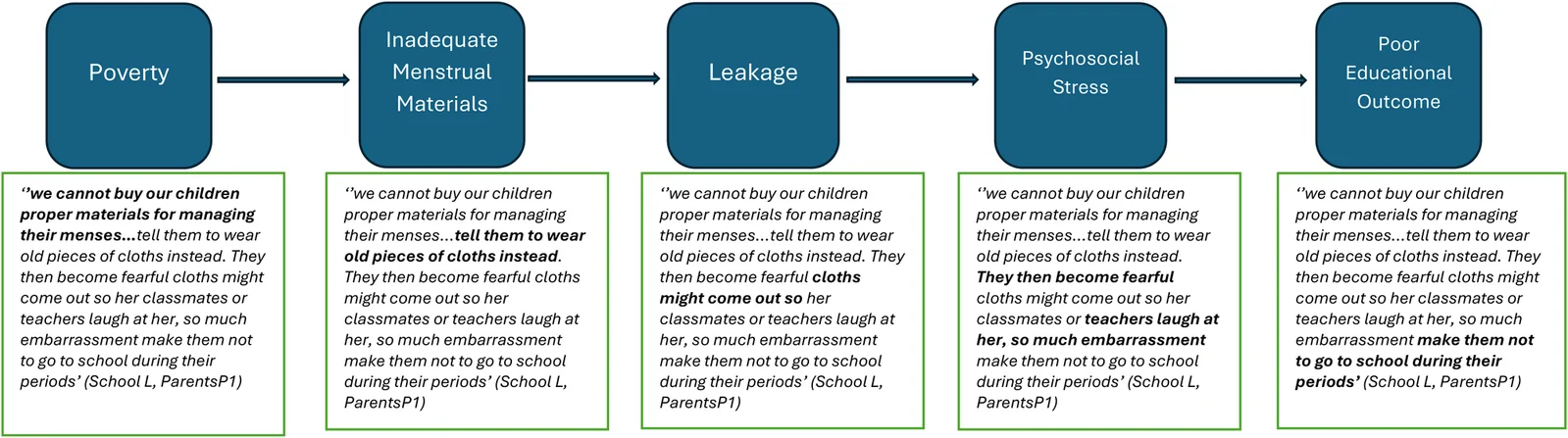
Full pathway 1.

#### Pathway 2: poverty to poor educational outcomes via inadequate menstrual materials and discomfort

This pathway began the same as pathway 1, with poverty leading to perceived proximal effects such as the inadequate or prolonged use of menstrual materials. However, participants stated that these proximal effects led to discomfort and pain from bruises, cuts, and chafing, adding a further dimension to the pathway (illustrated in Figure 3), with participants saying that this led to school absenteeism during their menses. However, we noted that only parents discussed this pathway, and they did not perceive it as causing psychosocial distress. Instead, they just linked it directly to poor educational outcomes (i.e., absenteeism). A few parents and teachers noted that menstrual pain, rather than discomfort from the materials used, was a critical reason for girls missing school; however, they did not perceive them as associated with any of the previously noted causal factors (poverty or inadequate menstrual materials).

**Figure 3:**
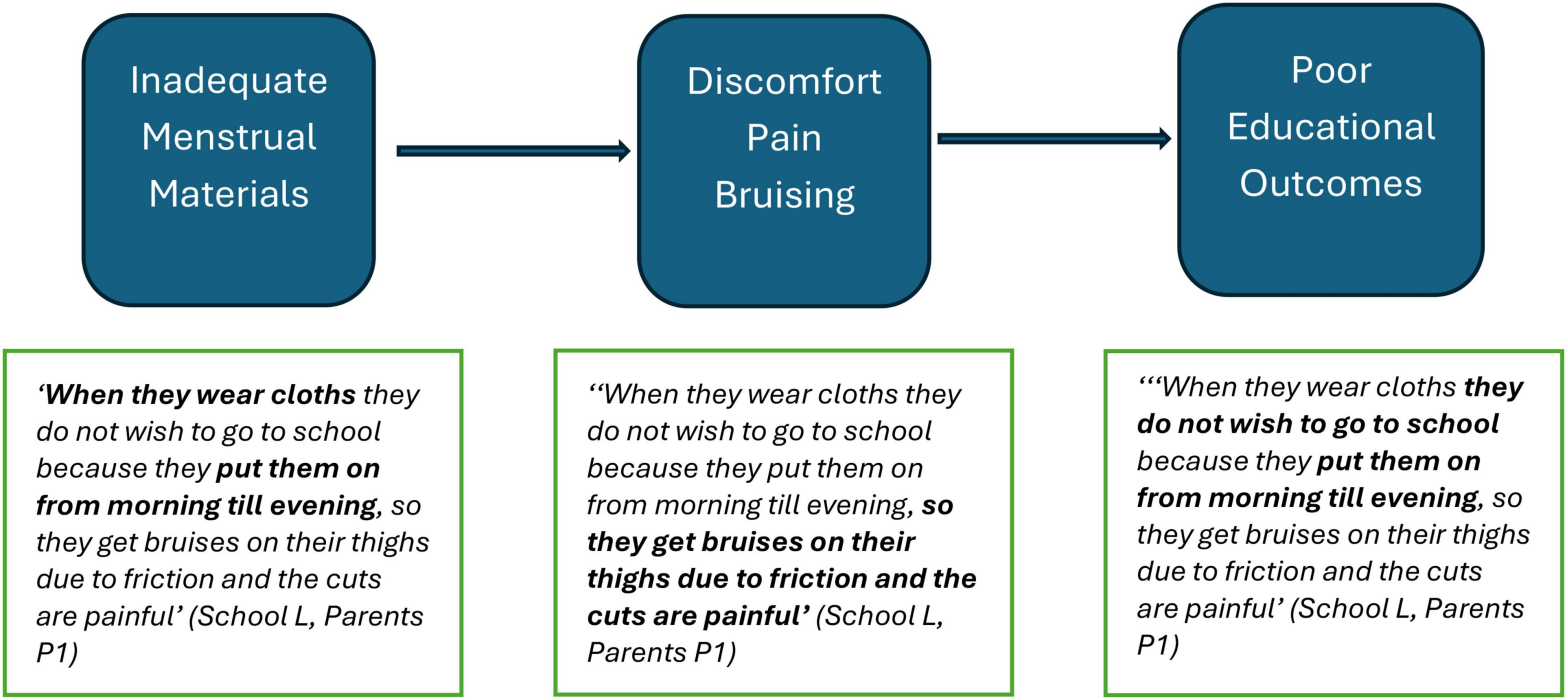
Pathway 2.

#### Pathway 3: inadequate menstrual materials to infections and poor educational outcomes

Another pathway highlighted by participants connected poverty to the inadequate and/or prolonged use of menstrual products (as illustrated in Figure 4). This was then perceived to drive a more distal outcome on this pathway: non-sexual RTIs. While RTIs were predominantly the outcome in this pathway, a single teacher noted that girls may lose focus in school due to feeling unwell from infections resulting from the prolonged use of menstrual materials. Only one girl associated STIs with sexual activity, and no teacher or parent considered that any factor would lead to sexual engagement resulting in an STI. Repercussions or consequences of contracting STIs were not discussed.

**Figure 4:**
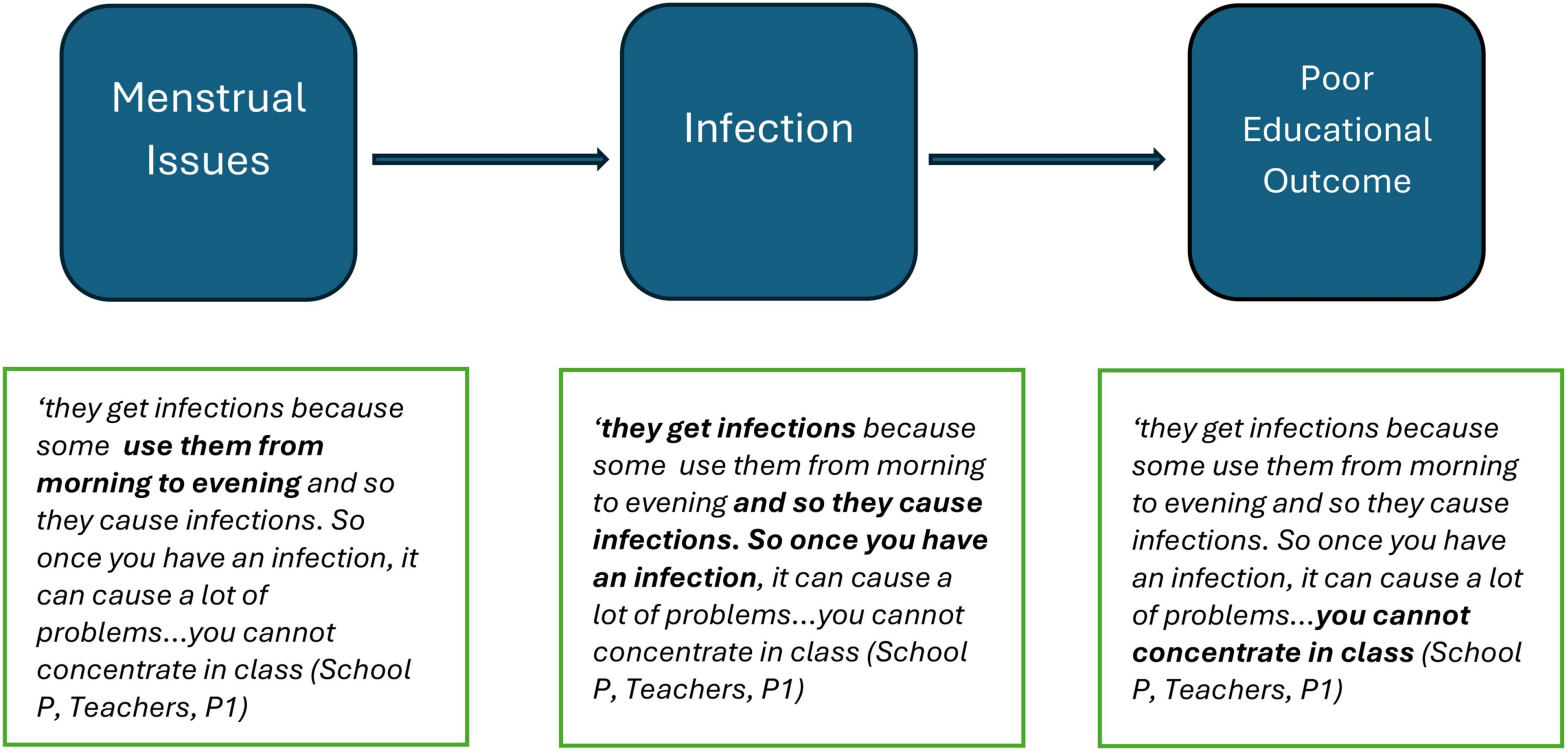
Pathway 3.

#### Pathway 4: poverty via inadequate menstrual materials to transactional sex

Pathway 4 was also perceived to begin with poverty as the causal driving factor. Participants frequently reported that they could not afford sanitary pads for their needs. This led to girls' engaging in transactional sex. Girls explained that peers engaged in transactional sex as a form of payment to obtain money for purchasing pads or to receive pads directly. Teachers and parents also identified that the inability to afford menstrual products drove girls into transactional sex to obtain them. The strong pathway, identified by all participant cohorts (Figure 5), terminated at this point, as no girls directly associated transactional sex with pregnancy. Only a single teacher and a single parent noted that the inability to afford menstrual products or lack of money led distally to pregnancy, without explicitly noting sexual activity/transactional sex as an intermediate causal “node”.

**Figure 5:**
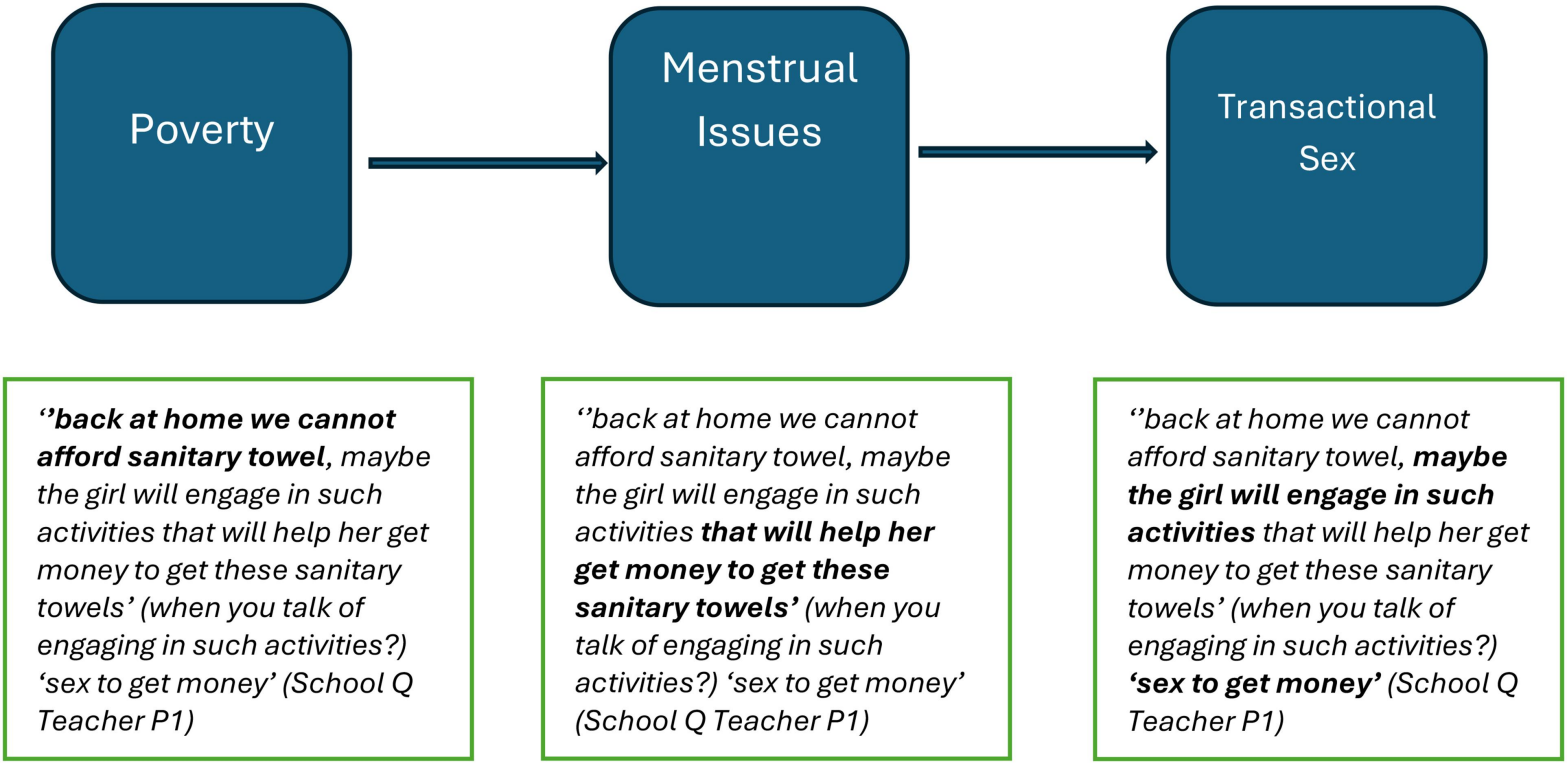
Pathway 4.

Separately, girls and teachers recognised that becoming pregnant would result in school dropout, although parents did not mentioned this. However, no participant directly associated this link with a menstrual-related cause, i.e., transactional sex in return for pads.

### Consolidated causal pathway model

A consolidated causal pathway was built using the above perceived causal associations made by girls, teachers, and parents (Figure 6). Notably, certain associations were voiced by more participants than others, with teachers more likely to notice effects on school outcomes. Where few participants reported any causal and outcome associations, these have been allocated as “weaker” (thinner dashed lines) when building this final causal pathway model. Participant quotes frequently linked the intermediate driver (node) of the pathway, with few participants describing a relationship from the key driver to the end outcome. Poverty was identified as the key driver of menstrual health and hygiene issues, with school absenteeism the most frequently reported outcome on the causal pathway. Although pregnancy and RTIs were also perceived as outcomes, these were identified by fewer participants and demonstrated a weaker association with menstrual hygiene issues. These were also rarely identified by participants as leading to a poor educational outcome. Poor educational outcomes, namely, school absenteeism, were therefore identified as the primary endpoint of the perceived menstrual health and hygiene causal pathway.

**Figure 6:**
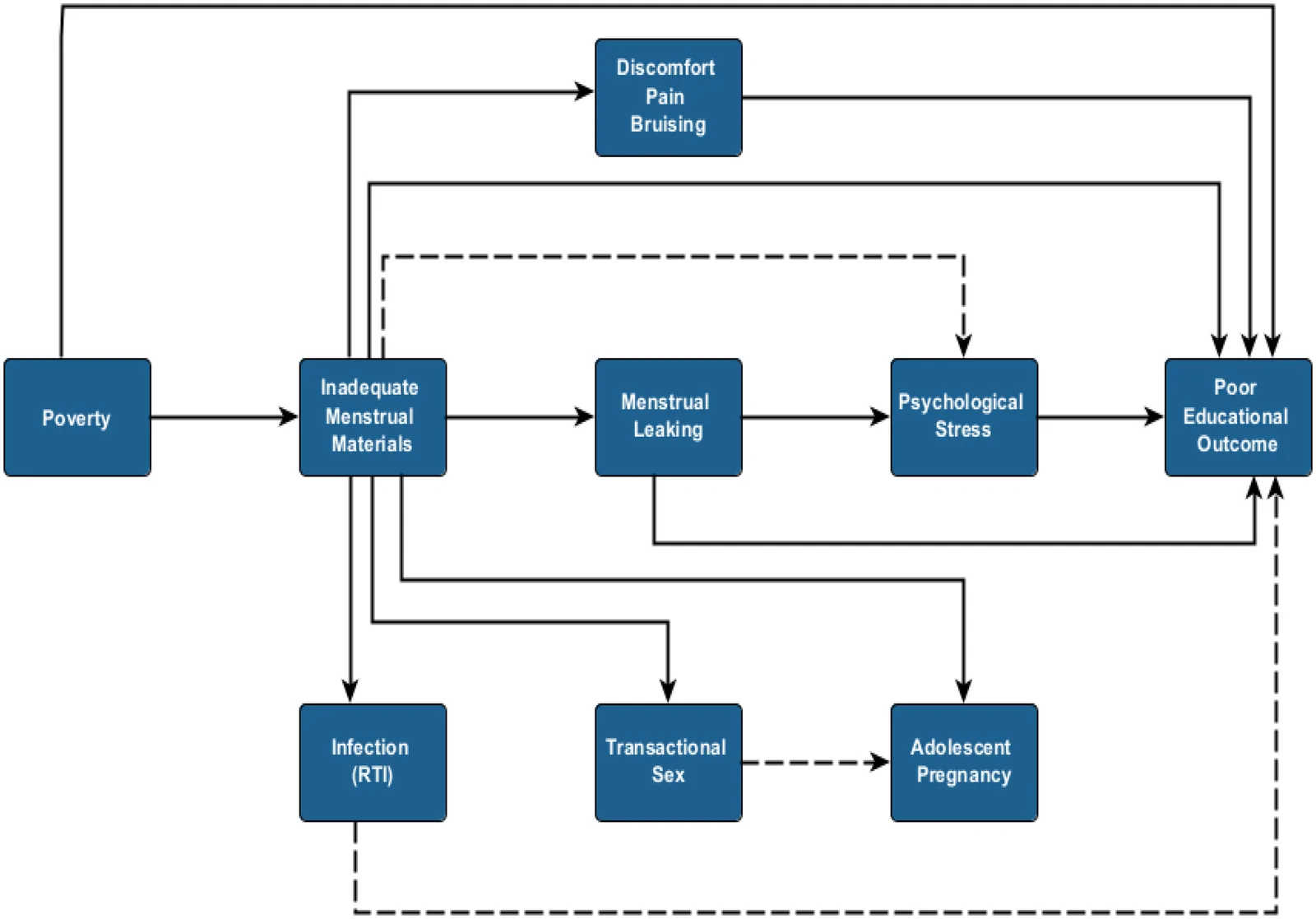
Consolidated causal pathway.

## Discussion

This study used data generated from FGDs among girls, teachers, and parents to explore perceived drivers of poor MHH and their impacts on girls' education and health, from which we modelled a causal pathway framework. Although the FGDs were not originally designed to build a causal pathway, this study found strong perceived associations with participants recognising cause-and-effect relationships. These perceived pathways can pinpoint where stakeholders believe interventions are most needed to reduce harmful outcomes.

Components of pathway 1 (poverty leading to poor educational outcomes via inadequate menstrual materials, leakage, and psychosocial distress) have been evidenced widely in the literature to date. Poverty, a widely documented phenomenon driving poor MHH, was recognised by our participants as the key driver. Studies in both low- and high-income countries report a lack of resources for sufficient quantities and quality of MHH materials (16, 26–28), resulting in a series of cascading effects. Qualitative and cross-sectional studies report that girls lack appropriate menstrual absorbents, with some using rags or other impromptu materials. Such studies have found an association between poor-quality materials and leakage or the fear thereof, as reported by our participants. Similarly, they report that girls feel embarrassed, ashamed, and stressed as a consequence (11, 16, 26, 29, 30). Such effects are compounded when teased by classmates or even teachers. Girls either absented themselves from school until their menses is over or remained in attendance while experiencing disengagement, diminshed active participation, or compromised concentration. In our study, both phenomena were perceived, although with a stronger focus on school absence.

School dropout due to menstrual-related factors may represent a cumulative process driven by repeated absence, resulting in disengagement or poor grades, or a wearing down of a girl’s psyche over time, rendering it less observable to others. A few studies consider dropout to be an indirect consequence of poor MHH mediated by repeated absenteeism (31), rather than a direct outcome. Conversely, Shah and colleagues interviews with schoolgirls revealed that poor MHH led to absence, but it was not a reason for dropout (32). One mixed-methods study asked girls opinions about “other” girls who had dropped out of school, finding that a high proportion attributed dropout to MHH-related causes, such as a lack of absorbent materials and embarrassment (33). We recommend that menstrual studies include girls who have dropped out of school to better represent this missed association with MHH.

We noted that, while odour was mentioned by parents and teachers (although not by girls) as an outcome of inadequate materials, participants did not link this further to the causal pathway. While fear of body odour was frequently cited by schoolgirls in qualitative interviews (32), their quantitative survey reported that only 2% cited it as a reason for missing school.

Pathway 2 also began with poverty as the perceived driver, resulting in the use of inadequate menstrual products. However, participants identified that wearing these products caused chafing, bruising, and discomfort. It was this resulting physical discomfort that led girls to report absenteeism from school. Limited studies corroborate these findings; Hennegan and colleagues reported in Uganda that discomfort affected girls' ability to attend school or participate in activities (34), while a study conducted in India noted that discomfort and irritation affected school participation and concentration (30). The weaker body of evidence in comparison suggests that, as with our pathway 2, physical discomfort as a perceived cause of absence or a lack of engagement is not as strong a driver as leakage, nor it is likely to lead directly to school dropout (although it may be part of a cumulative effect, as described above).

Painful periods (dysmenorrhea) were noted by a few teachers and parents as a reason for absence, although they were emphasised far less than the discomfort arising from poor materials. This finding might seem surprising given the substantial global evidence base identifying dysmenorrhea as a prevalent driver of school absenteeism. Research in Uganda indicates that four in five girls state menstrual cramps as a cause of missed schooling (35), while studies in India show that one-third of girls identify menstrual pain as a cause of school absenteeism (30). Numerous other studies also identify this issue (32, 33, 36, 37). Our emphasis on menstrual hygiene issues as our research aim may have led participants to focus more on MHH than on other causes of school absence. Dysmenorrhea may emerge as a strong driver within a general causal pathway, should a similar study be repeated with a wider research aim.

Pathway 3 was generated from participants' perceptions of how poor-quality products and their prolonged use were believed to have caused their infections. These were vague descriptions, however, with few specific symptoms mentioned. Some infections may have been caused by localised wounds resulting from irritation or friction. Others may signify an RTI, notably BV, which is associated with the use of poor-quality sanitary pads or “usual practices” that disrupt vaginal flora (6, 38, 39). Lower BV rates were reported in India among women who changed menstrual materials more frequently (40). Although Shah and colleagues (32) found that girls reporting symptoms of reproductive or urinary tract infections were more likely to miss school, they could not establish a direct causal relationship and recommended that future studies include clinical measures to better assess associations between infection and school absenteeism.

Engaging in transactional sex to obtain money for purchasing pads or to receive pads directly—the perceived key driver for pathway 4—was recognised across all groups as a consequence of an inability to afford enough disposable sanitary pads for the duration of menses. All participant groups agreed that girls may engage in transactional sex to obtain menstrual pads when they cannot afford enough supplies. Although girls often attributed this behavior to something “other” peers do, suggesting stigma, previous research and local studies confirm that “sex for pads” is relatively common within this setting (11, 16). Secondary school girls also identified pressure from parents, peers, and boda boda drivers as contributing factors to transactional sex (19). Despite recognising the connection between pad shortages and transactional sex, few participants linked this behaviour to unwanted pregnancy and school dropout, even though adolescent pregnancy causes many girls in Kenya to leave school each year (41).

As one in five Kenyan girls aged 15–19 years becomes pregnant or a mother, with the adolescent population accounting for more than 18% of births (42, 43), it remains unclear why this was rarely cited as an outcome by our participants. It may reflect the stigma attached to “visible” evidence of transactional sex (41) or a limited awareness of the risks associated with transactional sex (44, 45). Parents and girls may avoid contemplating these potential harms or consider them too distal and thus are unable to relate this to proximal issues around obtaining pads. Alternatively, they may have been reticent to discuss pregnancy as an outcome due to shame (46, 47). Irrespective of the underlying mechanism, it is concerning that this outcome was not prominent in participant narratives. A community component may be needed to resolve issues around MHH and the perceived need to prevent downstream effects, such as pregnancy and infection risk.

We note that girls' narratives mainly associated the proximal effects of poverty with poor MHH, less frequently connecting them to distal outcomes such as school dropout. We perceived parents and teachers to be a little more insightful than girls in their understanding of cause-and-effect relationships. These findings highlight the unique value and insight offered by incorporating both teacher and parent' perspectives in the knowledge and understanding of girls' menstrual health and poor outcomes (26, 48). We recommend including teachers or parents as participants in schoolgirl MHH studies.

Some limitations to this study warrant consideration. As inherent to data generated from FGDs, there may be bias if participants conform to the views of the group, or a social desirability effect to please the moderator. Any dominant personalities may overshadow the views of the quieter voices. However, we believe that the effects were minimal, given the repetition of the findings across groups. Our qualitative data were generated as a baseline for a pilot intervention study and were not designed specifically to elicit perceptions on causality. In-depth probing of associations did not occur, reducing the extent of these findings. Our methods to develop a causal pathway involved using only quotes that included both a perceived cause and a perceived outcome. Excluded narratives focusing solely on a singular event that had no linkage with either a cause or an outcome may have reduced the strength of perceived problems associated with girls facing menstrual challenges. We note that, stemming from qualitative discussions, the resultant pathways do not imply causality but only participants’ perceived pathways. We also note that discussions around transactional sex, STIs, pregnancy, and behavioural norms are complex and personal; consequently, information from girls and parents may have been muted to avoid peer judgement during the FGDs (49). In addition, while the original study was completed a decade ago, insights from our more recent research and evidence from other studies indicate that very little has changed (13, 19, 50). Products remain too expensive (51), with the provision of free pads patchy, inconsistent, or failing to meet individual needs (Nkansah et al. 2026). WASH facilities continue to be of poor quality (52, 53); while education on menstruation and puberty is progressing in some areas, this is not occurring systematically across all schools or countries (54, 55). Therefore, these experiences and perceptions remain valuable and relevant.

## Conclusion

Four perceived pathways linking poverty and poor MHH to an endpoint were used to develop a single causal pathway. The most frequently perceived outcome was school absenteeism, with pregnancy and school dropout noted much less frequently. As poverty, the perceived underlying cause in this pathway, is a complex target, focusing interventions on proximal perceived key nodes in the pathway, such as poor MHH, would be valuable. The development of a causal pathway is useful for identifying the perceptions of relevant parties and for understanding points to employ interventions. Our perceived causal pathway provides a platform for policy and programme development to improve menstrual health and well-being for girls in Kenya, reducing the harmful outcomes and risks associated with poor MHH.

Future research should include out-of-school girls to better understand how MHH links to school dropout rates versus temporary and cumulative absence. In addition, studies utilising clinical measures are needed to evaluate the relationship between infections and school absenteeism. Parents and teachers should be considered as key informants in adolescent MHH studies. Most importantly, future causal pathway research must address the broader MHH framework, specifically integrating WASH facilities, menstrual products, education, and supportive policies.

## Data Availability

The raw data supporting the conclusions of this article will be made available by the authors, upon reasonable request.
